# Supplementary material for: Development and deployment of a public mid‐density genotyping panel for crimson clover (Trifolium incarnatum L.) based on whole‐genome re‐sequencing of diverse germplasm
Source: Plant Genome. 2026 Sep 29;19(4):e70297. doi: 10.1002/tpg2.70297 (PMC13624372; doi:10.1002/tpg2.70297)
Supplement: Supplementary file 1 [file TPG2-19-e70297-s001.pdf]

Table S1. Spearman correlation between WGR mapping depth and enrichment panel probe density across 1-Mb genomic windows.

Parameter	Value
**Analysis**	
Method	Spearman rank correlation
Genomic windows	410
Window size	1 Mb (non-overlapping)
Chromosomes	7 (NC_060059.1-NC_060065.1)
Reference genome	GCF_020283565.1 (ARS_RC_1.1)
Depth computation tool	mosdepth v0.3.10
Probe intersection tool	BEDTools intersect
Enrichment panel	Auburn Crimson Clover 30K
**Genotypes**	
Total genotypes	194
High-depth (~50X, SNP discovery)	45
Low-pass (~2X, diversity panel)	149
**Mapping rates (50X panel, n = 45)**	
Mean ± SD	79.51 ± 3.73%
Range	62.49-93.16%
**Spearman correlation**	
ρ	0.333
95% CI (Fisher z)	[0.244, 0.416]
p-value (asymptotic)	< 0.001
Permutation p (9,999 permutations)	< 0.0001
Variance explained (ρ^2)	11.1%
Trimmed ρ (3×IQR outliers removed)	0.374
Trimmed n	392
Trimmed p-value	< 0.001
**Probe count per window**	
Minimum	10
Q1	46
Median	53
Q3	57
Maximum	245
Mean ± SD	50.8 ± 14.7
Windows with zero probes	0

Table S2. Per-chromosome summary of WGR mapping depth and enrichment panel probe density.

Chromosome	Label	Windows (1 Mb)	Size (~Mb)	Total Probes	Mean Probes/ Window	Min Probes	Max Probes	Mean Depth (X)	SD Depth	CV Depth (%)
NC_060059.1	Chr1	54	54	2,848	52.7	26	89	17.93	9.81	54.7
NC_060060.1	Chr2	75	75	3,607	48.1	10	102	11.50	3.94	34.3
NC_060061.1	Chr3	55	55	2,928	53.2	18	91	12.89	4.27	33.1
NC_060062.1	Chr4	61	61	3,096	50.8	14	95	12.61	3.88	30.8
NC_060063.1	Chr5	58	58	2,621	45.2	11	88	12.15	4.01	33.0
NC_060064.1	Chr6	47	47	2,648	56.3	19	245	13.48	4.53	33.6
NC_060065.1	Chr7	60	60	3,074	51.2	15	93	13.03	4.12	31.6
Total/Mean		410	~410	20,822	50.8	10	245	13.54		

Probe counts represent 20,818 merged capture regions on the seven primary chromosomes. The full panel comprises 28,867 unique probes synthesized by Twist Bioscience; multiple probes may tile a single target region, and adjacent targets are merged during manufacturing, resulting in fewer merged regions than individual probes.
